# The ChatGPT source cue shapes answer acceptance in a reasoning task

**DOI:** 10.3389/fpsyg.2026.1923236

**Published:** 2026-10-23

**Authors:** Rosa Angela Fabio, Elisabetta Rantuccio, Francesca Bruno

**Affiliations:** 1 Department of Biomedical, Morphological and Functional Imaging Sciences, University of Messina, Messina, Italy; 2 Department of Cognitive Science, University of Messina, Messina, Italy

**Keywords:** ChatGPT, cognitive conflict, Cognitive Reflection Test, critical thinking, evaluative judgment, source attribution

## Abstract

**Introduction:**

As generative AI systems increasingly provide answers in everyday and academic contexts, understanding how source attribution influences evaluative judgment has become critical.

**Methods:**

In a within-subjects design, 103 participants evaluated 30 pre-generated reflective-correct, intuitive-incorrect, and arithmetic control-correct answers labeled ChatGPT or computer. Assignment of two parallel batteries to source labels and trial presentation order were randomized, and displays were visually matched. An independent unlabeled pretest with 35 participants supported battery comparability.

**Results:**

Participant-level analysis showed greater overall acceptance under ChatGPT (68.0% versus 63.4%, *p* = 0.002). A trial-level logistic mixed model with crossed participant and parallel-problem-pair random intercepts showed a source-by-category interaction (*p* < 0.001): acceptance was higher under ChatGPT for arithmetic controls (OR = 6.08, 95% CI [2.70, 13.69]), while source contrasts within the two CRT categories were not significant. Judgment accuracy is reported descriptively. Signal-detection analyses of CRT answers detected no significant source difference in sensitivity (*p* = 0.930) or criterion (*p* = 0.082). Evaluation times were longer under ChatGPT in the raw-time analysis (*p* = 0.034), but the source difference was not significant after log transformation (*p* = 0.180). Exploratory correlations detected no significant associations between source-related differences and trust, AI attitudes, or critical-thinking disposition.

**Discussion:**

These findings distinguish overall source-related endorsement, particularly for arithmetic controls, from successful discrimination between correct and incorrect CRT answers.

## Introduction

As generative AI systems such as ChatGPT become increasingly integrated into academic, professional, and everyday decision-making contexts, individuals are frequently required to evaluate externally provided answers rather than generate solutions themselves. In such contexts, judgments are shaped not only by informational content but also by source-related cues, including perceived competence, authority, and credibility.

From a dual-process perspective, evaluative judgment can be understood as involving the interplay between rapid intuitive impressions and more deliberate evaluation. Cognitive conflict paradigms, such as the Cognitive Reflection Test (CRT), provide a well-established framework for examining this interplay because they present intuitively compelling but incorrect responses that must be rejected in favor of reflective solutions. This framework motivates the study of answer evaluation, while the categories of supplied answers remain distinct from measurements of participants’ processing modes (Frederick, 2005; Evans, 2008; De Neys and Pennycook, 2019).

In many judgment and decision-making contexts, individuals evaluate externally provided information rather than generate responses themselves. In these situations, source-related cues such as credibility, authority, and expertise often guide evaluative decisions. Research on heuristic and systematic processing shows how source and message cues can contribute differently to persuasion (Chaiken, 1980). Applied to answer evaluation, this distinction suggests that endorsing an apparently credible source and verifying the validity of its answer may involve different judgments.

In the context of generative AI, expectations about technological competence may amplify the contribution of source-related cues to evaluative judgment. Evidence on trust in human–robot interaction highlights the importance of system and task characteristics (Hancock et al., 2011), while algorithm appreciation studies show that people can favor algorithmic advice over human judgment (Logg et al., 2019). Broader work on large language models and their use in human–computer interaction research situates these questions within a rapidly expanding field (Tabone and de Winter, 2023; Teubner et al., 2023). These perspectives motivate examining whether an AI source cue is associated with acceptance and how users allocate attention during evaluation.

A central construct in human–AI interaction is trust. Research on automation emphasizes that reliance should be calibrated to system capabilities and task demands (Dzindolet et al., 2003; Lee and See, 2004; Gulati et al., 2019). A related metacognitive literature examines how ease of processing can inform judgments: perceptual fluency can affect perceived truth, and processing fluency contributes to a range of evaluations (Reber and Schwarz, 1999; Alter and Oppenheimer, 2009). Source attribution and fluency are distinct cues. In the present task, the visual format was matched, whereas the source label and corresponding icon varied; fluency therefore provides theoretical background for credibility judgments, while the experimental comparison concerns source attribution.

This literature also contains effects in the opposite direction. Dietvorst et al. (2015) found that observing algorithmic errors can reduce willingness to use algorithmic forecasts. Longoni et al. (2022) reported lower perceived accuracy for news attributed to generative AI than to humans. Altay and Gilardi (2024) found that labeling headlines as AI-generated reduced perceived accuracy and willingness to share them, including when the headlines were true or human-created. Together with algorithm appreciation, these results suggest that the direction of a source effect depends on the task, comparison source, and experience with system performance. A numerical answer-evaluation task therefore provides a specific test within a literature that includes both acceptance and skepticism.

Recent empirical work has examined AI-based chatbot interactions in relation to critical-thinking skills (Fabio et al., 2025b), extending interest from general attitudes to users’ engagement with AI-assisted information. Such interaction studies address a broader setting than source attribution alone: dialogue, answer content, and interaction history may all contribute to evaluation. Research on general AI attitudes further distinguishes favorable views of AI benefits from concerns about its risks (Schepman and Rodway, 2020, 2023). These distinctions motivate asking whether endorsement in a controlled labeling task is associated with trust and attitudes, and whether evaluative behavior varies with critical-thinking disposition.

From a dual-process perspective, evaluative judgment can involve conflict between intuitively compelling responses and analytically supported alternatives. Dual-process models distinguish automatic and controlled contributions to reasoning, while contemporary accounts also examine the possibility of rapid logical responses (Evans, 2008; Kahneman, 2011; De Neys and Pennycook, 2019; De Neys, 2021). Thus, response correctness and elapsed time do not uniquely classify the underlying process. In an evaluative task, the behavioral question concerns which answer is accepted as correct, particularly when the problem admits a compelling but incorrect alternative. The terms reflective and intuitive identify the supplied solution categories used here.

Thus, the present study conceptualizes ChatGPT attribution as a potential source cue that may influence evaluative judgment under conflict, rather than as a simple credibility enhancer. This view is consistent with contextual accounts emphasizing dynamic shifts between controlled and automatic processing as a function of task demands and informational cues (Fabio et al., 2019).

Evaluative judgment under cognitive conflict is shaped not only by task characteristics and informational cues, but also by individual differences. Studies of misinformation associate analytic thinking with reduced susceptibility to false news (Bronstein et al., 2019; Pennycook and Rand, 2019). Analytic reasoning performance and a disposition toward critical thinking are related research topics but distinct constructs. In AI-mediated contexts, critical-thinking disposition may be associated with more selective evaluation of authoritative or plausible information. Examining it alongside source-labeled judgments therefore addresses whether contextual endorsement varies with an evaluative orientation.

Critical-thinking attitudes capture a disposition toward systematic analysis, search for truth and openness, analyticity, and inquisitiveness (Fabio et al., 2025a). Such attitudes may support evidence evaluation, consideration of alternatives, and suspension of premature judgment. They are assessed separately from performance in the present task, allowing us to explore whether this disposition is associated with source-related differences in acceptance and evaluation latency.

The present study examined whether ChatGPT attribution, compared with a generic Computer label, was associated with different evaluations of intuitive and reflective answers. Drawing on dual-process and contextual theories, we used CRT-derived problems containing normatively correct solutions and compelling incorrect alternatives. Participants judged pre-generated answers rather than solving the classic CRT independently. The principal research question therefore concerns source attribution during numerical answer evaluation.

By presenting reflective-correct, intuitive-incorrect, and control-correct answers under the two source labels, the study examined acceptance and latency within a common response format. The Computer condition represents another technological source, which may itself evoke expectations of arithmetic reliability. The comparison consequently concerns a named conversational AI source and a generic computer source; it differs from comparisons with human experts or with unlabeled answers. Answer category and answer truth were not fully crossed, because the reflective and control answers were correct and the intuitive answers were incorrect.

Acceptance indicates whether a participant judges a supplied answer correct, irrespective of its actual truth. Judgment accuracy indicates whether that evaluation matches the mathematical answer key: accepting a correct answer and rejecting an incorrect answer are both accurate judgments. Signal-detection analysis complements these measures by distinguishing sensitivity to correct versus incorrect CRT answers from the criterion for responding “correct”. This distinction is essential because greater acceptance can apply to both accurate and inaccurate information.

We predicted that answers attributed to ChatGPT would be accepted more often than answers attributed to Computer. Response times were recorded as evaluation latencies and examined exploratorily, together with source-by-category interactions and signal-detection measures. Longer latency could reflect scrutiny, uncertainty, attention to source information, or hesitation. Considering acceptance and latency together characterizes how participants evaluate supplied answers, while keeping the theoretical interpretation of underlying processes separate from the measured outcomes.

## Materials and methods

### Participants

A total of 103 participants (67 women, 36 men) took part in the study. Mean age was 25.33 years (SD = 7.01; median = 23; range = 18–63). Participants were recruited through convenience sampling and word of mouth. Fifty-one participants (49.5%) were university students, including one who also worked as a bartender; two (1.9%) were doctoral students, including one who also taught. Forty-two (40.8%) reported other occupations, covering education, healthcare, administration, retail and hospitality, technical work, and public services. Seven (6.8%) reported no current occupation and one (1.0%) was retired.

Before participating, all individuals received information about the study and provided informed consent electronically by selecting an affirmative consent option. Participation was voluntary. Data were recorded under participant codes.

The sample size was determined pragmatically; no preregistered *a priori* power analysis was conducted. As an approximate sensitivity benchmark for a single two-sided Pearson correlation, *N* = 103, *α* = 0.05, and 80% power correspond to |*r*| ≈ 0.27 using a Fisher-*z* approximation. This benchmark concerns the exploratory correlations. The within-subjects design compares source conditions within the same participants, reducing variation associated with differences in baseline performance.

### Materials and measures

#### Cognitive reflection task

The experimental task was based on a modified version of the Cognitive Reflection Test (CRT; Frederick, 2005), a well-established paradigm designed to investigate the tension between intuitive and reflective reasoning. CRT problems are characterized by an intuitive–reflective conflict, in which an immediately compelling response must be inhibited in favor of a more deliberative solution. This paradigm is widely used to examine the interaction between intuitive (System 1) and reflective (System 2) processing (Evans, 2008; De Neys and Pennycook, 2019; De Neys, 2021).

A classic illustration of this dissociation is provided by the well-known bat-and-ball problem. When asked, “A bat and a ball cost €1.10 in total. The bat costs €1.00 more than the ball. How much does the ball cost?”, many individuals intuitively respond €0.10. However, this intuitive answer is incorrect, as a reflective analysis reveals that the correct solution is €0.05. This example highlights how a compelling incorrect alternative can compete with the mathematically correct solution.

In the present study, the CRT paradigm was adapted to examine how attribution of a response to an artificial intelligence system influences evaluative judgment. Rather than asking participants to generate their own answers, each trial presented a problem paired with a pre-generated response, which participants were asked to judge as correct or incorrect, yielding a measure of answer acceptance.

For example, instead of solving the bat-and-ball problem themselves, participants were shown the problem together with a response such as “The ball costs €0.10” or “The ball costs €0.05” and were asked to indicate whether the provided response appeared correct or incorrect (Frederick, 2005). Examples of the response attribution manipulation and of the visual presentation of the task are shown in Figure 1. To minimize perceptual and interface-related confounds, the two attribution conditions were presented using visually matched displays; they differed only in the source label and the corresponding small icon.

**Figure 1:**
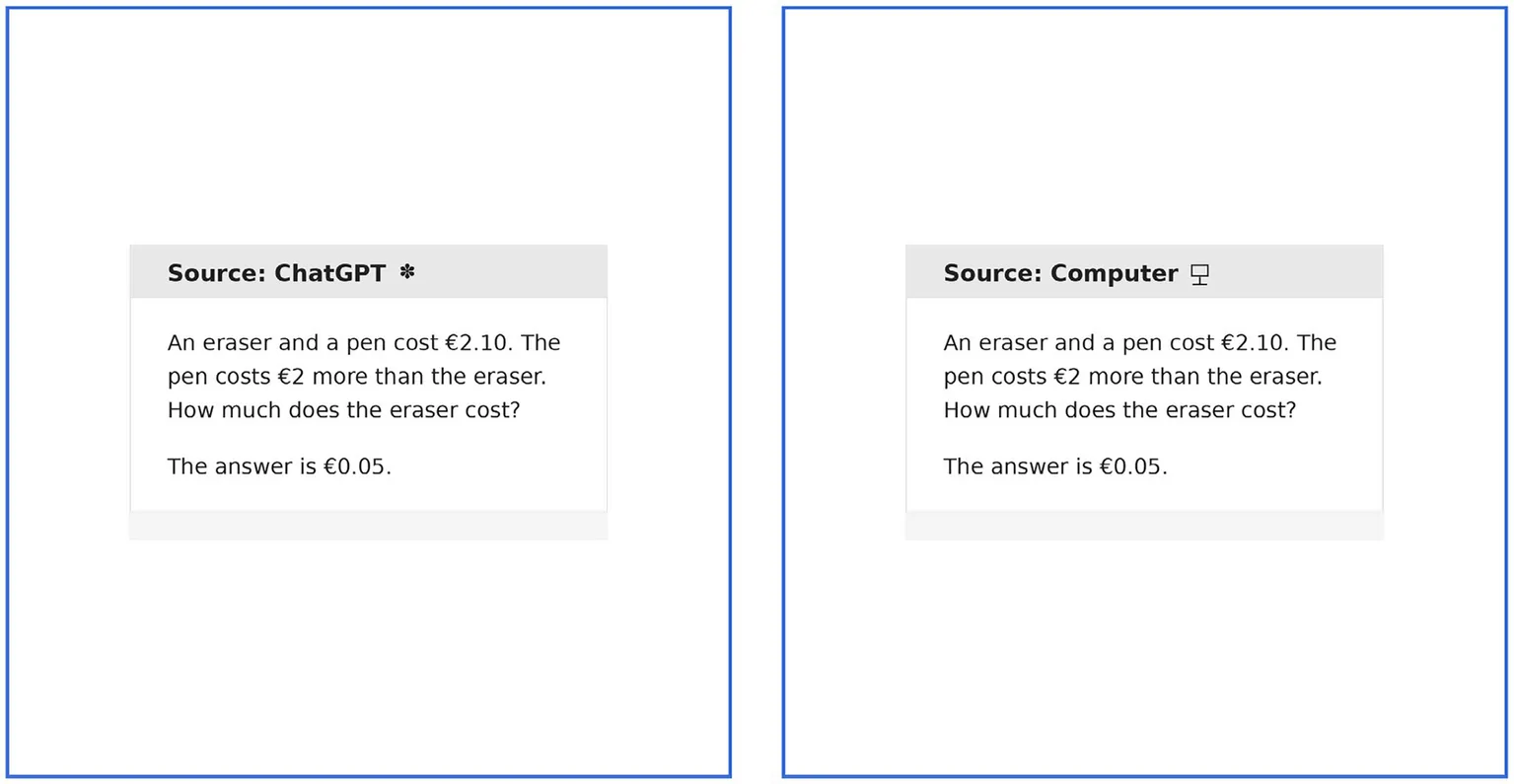
Example of stimulus presentation in the two attribution conditions. The figure illustrates an example of a CRT problem paired with a pre-generated response as presented to participants. The ChatGPT and Computer conditions were matched for layout, size, font, color, spatial position, and response format. The only difference between the two displays was the source attribution label (“Source: ChatGPT” vs. “Source: Computer”) and the corresponding small source icon. In both conditions, participants were asked to judge whether the response provided appeared correct or incorrect.

#### Trial structure and response categories

The task consisted of 30 trials, each presenting a problem and one pre-generated answer with either the ChatGPT or Computer source label. Fifteen trials carried each label, with five per label in each answer category. The two batteries comprised 15 pairs of parallel problems: corresponding forms shared the same reasoning structure but differed in numerical values and context. Assignment of the batteries to source labels was randomized between participants, and trial presentation order was randomized. Each participant saw each problem once; no identical problem was presented with both a reflective-correct and an intuitive-incorrect answer. The displays were visually matched and differed in source label and corresponding small icon. Computer denotes the displayed comparison source; the experiment manipulated attribution of fixed answers rather than comparing outputs obtained from live systems.

The initial instructions informed participants that they would see logical–mathematical problems accompanied by answers presented as generated or supplied by ChatGPT or a computer system. They were asked to judge each supplied answer as correct or incorrect. No further information about an AI model or computer software was provided. Source attribution during the task was conveyed by the source label and its associated icon. All answers were prepared in advance by the researchers.

Across trials, three categories of responses were presented (see Table 1):

(1) CRT trials with a reflective correct response, in which the provided answer corresponded to the normatively correct, reflective solution.
(2) CRT trials with an intuitive incorrect response, in which the provided answer corresponded to the typical intuitive but incorrect solution.
(3) Control trials (non-CRT), consisting of simple arithmetic problems without an intuitive-reflective conflict and paired with objectively correct responses.

**Table 1:** Distribution of trials across answer categories and source conditions.

Trial category	Total number of trials	Source (ChatGPT/computer)	Intuitive–reflective conflict	Example
CRT—reflective correct response	10	5/5	Yes	Eraser-and-pen → €0.05
CRT—intuitive incorrect response	10	5/5	Yes	Bat-and-ball → €0.10
Control (non-CRT)	10	5/5	No	3 apples + 4 apples → 7

Each source condition included five trials per answer category. The two batteries were counterbalanced across source labels between participants. Controls were simple arithmetic problems paired with correct answers. The full item list is provided in the Supplementary material.

Participants were not required to solve the problems. Their task was solely to judge whether the provided response appeared correct or incorrect, yielding a measure of answer acceptance.

#### Acceptance, objective answer correctness, and judgment accuracy

Objective answer correctness is the mathematical truth status of the supplied answer, established by the normative solution. Acceptance indicates the participant’s judgment: *A* = = 1 when the supplied answer is judged correct and *A* = 0 when it is judged incorrect. Judgment accuracy indicates whether that evaluation matches the answer key. If *T* = 1 denotes an objectively correct answer and *T* = 0 an incorrect answer, accuracy is *Y* = TA + (1 − *T*)(1 − *A*). Accordingly, *Y* = *A* for reflective-correct and control-correct answers, and *Y* = 1 − *A* for intuitive-incorrect answers. Accepting a correct answer and rejecting an incorrect answer are therefore both accurate judgments. Because accuracy is derived from the same judgments as acceptance, it is presented descriptively; inferential analyses focus on acceptance, evaluation latency, and signal-detection measures.

#### Control of item difficulty

An independent sample of 35 participants evaluated both batteries in a pretest without source attribution: no Computer or ChatGPT context was provided. Correctness in the pretest refers to judgment accuracy, namely accepting a correct answer or rejecting an incorrect answer. Mean accuracy for reflective answers was 0.510 in the first battery and 0.504 in the second; for intuitive answers, it was 0.620 and 0.650, respectively. Paired comparisons used the standard deviations of the within-participant differences (reflective: SDdiff = 0.484; intuitive: SDdiff = 0.490). Neither comparison was significant: reflective, *t* (34) = 0.073, *p* = 0.942; intuitive, *t* (34) = −0.362, *p* = 0.719. Scores were strongly correlated across batteries for reflective (*r* = 0.93) and intuitive answers (*r* = 0.89), both *p* < 0.0001. Control accuracy was 100% in both batteries (*M* = 1.00, SD = 0), with identical individual scores. The small mean differences, strong correlations, and identical control performance support battery comparability. Assignment of the batteries to source labels was counterbalanced between participants.

#### Procedure and recording

Each trial recorded a binary correct/incorrect judgment and its response time, measured from presentation of the answer to the judgment. Judgment accuracy was derived from the answer key. RTs below 300 ms or above 60,000 ms were excluded from latency summaries. Of the 3,090 possible RT observations, 109 (3.53%) fell outside these thresholds: one below 300 ms and 108 above 60,000 ms. One additional RT was missing (0.03%), leaving 2,980 observations (96.44%). Excluded or missing observations by category were 36 reflective, 30 intuitive, and 3 control under ChatGPT, and 22 reflective, 14 intuitive, and 5 control under Computer. All 103 participants contributed valid RTs to every source-by-category cell. RT screening did not remove the corresponding valid binary judgments from the acceptance analysis.

Acceptance and judgment accuracy were summarized as participant-level proportions within each source-by-category cell, with five judgments per cell. RTs were averaged within cells after screening. The analysis dataset contained 103 participants with all 30 judgments. One nonbinary entry in the reflective ChatGPT judgments was corrected to 1 following confirmation of the original response. All behavioral estimates reported here use that corrected value.

#### Self-report questionnaires

##### Human-Computer Trust Measure (HCTM)

Trust in artificial intelligence systems was assessed using the Human-Computer Trust Measure (HCTM; Gulati et al., 2019). The HCTM is a self-report questionnaire developed to measure trust toward computer-based and AI-related systems. In the present study, the first three negatively worded risk/caution items were reverse-coded before computing the total score, so that higher scores indicated greater trust in AI. Internal consistency was good (Cronbach’s alpha = 0.87).

##### General Attitudes toward Artificial Intelligence Scale (GAAIS)

General attitudes toward artificial intelligence were measured using the General Attitudes toward Artificial Intelligence Scale (GAAIS; Schepman and Rodway, 2020). The GAAIS comprises two subscales measuring positive attitudes (e.g., perceived usefulness and benefits of AI) and negative attitudes (e.g., concerns about risks, loss of control, or societal impact). Subscale scores were computed separately, with higher scores reflecting stronger positive or negative attitudes toward AI, respectively. Internal consistency was high for the positive-attitudes subscale (Cronbach’s alpha = 0.91) and satisfactory for the negative-attitudes subscale (Cronbach’s alpha = 0.82).

##### Critical Thinking Attitude Scale (CTAS)

Dispositional critical thinking was measured using the 26-item Critical Thinking Attitude Scale (CTAS; Fabio et al., 2025a). The scale addresses systematicity, search for truth and openness, analyticity, and inquisitiveness. It assesses an evaluative disposition rather than reasoning ability itself. The two negatively worded administered items (10 and 11) were reverse-coded on the five-point response scale before averaging the items, so that higher scores indicated a stronger critical-thinking disposition. Internal consistency for this scoring was Cronbach’s *α* = 0.87.

#### Overview of measures

Together, HCTM, GAAIS, and CTAS captured individual differences in trust toward AI systems, general AI-related attitudes, and critical thinking disposition. These measures were used in exploratory analyses to examine whether stable dispositional factors were associated with evaluative judgments during the experimental task or with attribution-related differences between the ChatGPT and Computer conditions.

### Design and procedure

The study used a within-subjects design with ChatGPT and Computer source labels and balanced trial counts across answer categories (Table 1). Assignment of the two pretested parallel batteries to source conditions was randomized between participants; trial order was also randomized. Administration took place online using PsyToolkit (Stoet, 2010, 2017). Participants provided informed consent and demographic information, completed the experimental task, and then completed the questionnaires. At the end of the study, participants were informed that the answers had been prepared by the researchers and that the ChatGPT and Computer labels had been assigned for experimental purposes.

### Data analysis

Trial-level answer acceptance was analyzed using a binomial generalized linear mixed model with a logit link, fixed effects of source label, answer category, and their interaction, and crossed random intercepts for participants and the 15 parallel-problem pairs (Jaeger, 2008). The model included all 3,090 binary judgments. Dataset columns recorded the source actually displayed; corresponding columns across conditions identified the same parallel-problem pair. The grouping factor therefore represented the shared problem structure across the two parallel formulations. Models were fitted by maximum likelihood using a Laplace approximation with glmer in lme4 version 1.1–35.1 in R version 4.3.3 (Bates et al., 2015). The bobyqa optimizer was used, with a second fit using nloptwrap to assess numerical agreement. A likelihood-ratio test compared models with and without the source-by-category interaction. Category-specific source contrasts are reported as odds ratios with Wald 95% confidence intervals and unadjusted two-sided *p* values; Holm adjustment across the three contrasts was checked and did not change the significance conclusions.

Model diagnostics included convergence codes and inspection of the estimated random-effect variances. Sensitivity fits removed any random intercept estimated at zero and added an independent random slope for source within participants. These checks evaluated whether the category-specific findings depended on the optimizer or on a random-effect component estimated at the boundary.

For comparison with the original participant-level analysis, binary judgments were also aggregated to proportions and analyzed using a 2 × 3 repeated-measures ANOVA, with Source label (ChatGPT, Computer) and Answer category (reflective-correct, intuitive-incorrect, control-correct) as within-subjects factors. Judgment accuracy was reported descriptively. RT analyses used participant-level cell means of screened raw times and, as a distributional sensitivity analysis, means of trial-level natural-log RTs. Mauchly’s test assessed sphericity for the three-level category factor and its source interaction; Greenhouse–Geisser corrections were applied when sphericity was rejected. The two-level source factor does not require this correction. Partial *η*^2^ was reported, with *α* = 0.05. Exploratory age-adjusted source comparisons regressed each participant’s overall ChatGPT-minus-Computer difference on an intercept and mean-centered age.

Exploratory Pearson correlations examined questionnaire scores and ChatGPT-minus-Computer differences in acceptance, judgment accuracy, and raw evaluation latency. Questionnaire items were averaged after the stated reverse scoring; positive and negative GAAIS scores were kept separate. Paired *t*-tests compared pretest accuracy across batteries, and Pearson correlations characterized their correspondence.

Internal consistency of the acceptance difference scores was estimated using the 15 matched parallel-problem pairs. For each pair, the Computer acceptance judgment was subtracted from the ChatGPT judgment; the mean of these differences reproduced the participant’s overall source-difference score. Cronbach’s alpha was computed across all 15 paired differences and separately within each five-pair category. For judgment accuracy, intuitive-answer differences were sign-reversed before computing alpha. These estimates concern behavioral acceptance and accuracy differences, separately from questionnaire internal consistency.

Signal-detection analyses were conducted separately for each participant and source condition, using reflective-correct CRT answers as signal trials and intuitive-incorrect answers as noise trials. Acceptance of a correct answer was a hit; acceptance of an incorrect answer was a false alarm. Arithmetic controls were excluded. The loglinear correction was applied uniformly: *H* = (hits + 0.5)/6 and *F* = (false alarms + 0.5)/6, with five trials of each type per source. Sensitivity was *d*′ = Φ^−1^(*H*) − Φ^−1^(*F*), and criterion was *c* = −[Φ^−1^(*H*) + Φ^−1^(*F*)]/2; negative c indicates a more liberal acceptance criterion. Paired two-sided *t*-tests compared *d*′ and *c* between source conditions, with 95% confidence intervals for ChatGPT-minus-Computer differences (Hautus, 1995).

A post-hoc sensitivity analysis assessed whether the overall acceptance difference persisted after excluding participants who incorrectly rejected more than 10% of the ten simple arithmetic control answers (at least two errors across both source conditions). We repeated the paired two-sided comparison of overall acceptance and the CRT sensitivity comparison in the restricted sample. The full sample remained the primary analysis sample. This exclusion used task performance rather than a dedicated attention check.

## Results

### Descriptive statistics

Table 2 and Figures 2–4 summarize answer acceptance, judgment accuracy, and evaluation latencies. Acceptance was higher under ChatGPT in all three categories. Judgment accuracy expresses whether each acceptance or rejection matched the answer key. Raw mean evaluation times were also longer under ChatGPT; the transformed-time analysis is reported separately below.

**Table 2:** Descriptive statistics for acceptance, judgment accuracy, and raw evaluation times by source label and answer category.

Response type	Attribution	Answer acceptance (*M*, SD)	Judgment accuracy (*M*, SD)	Response time (ms) (*M*, SD)
Reflective	ChatGPT	0.511 (0.258)	0.511 (0.258)	14,495.14 (9,176.47)
Reflective	Computer	0.474 (0.274)	0.474 (0.274)	13,215.40 (7,754.37)
Intuitive	ChatGPT	0.542 (0.237)	0.458 (0.237)	14,019.21 (8,472.48)
Intuitive	Computer	0.503 (0.250)	0.497 (0.250)	12,946.58 (7,103.88)
Control	ChatGPT	0.986 (0.051)	0.986 (0.051)	9,159.08 (5,479.85)
Control	Computer	0.924 (0.151)	0.924 (0.151)	8,382.10 (4,960.56)

Acceptance and judgment accuracy are proportions; RTs are milliseconds. Accuracy equals acceptance for correct answers and 1 − acceptance for incorrect answers. Computer denotes the displayed comparison source label.

**Figure 2:**
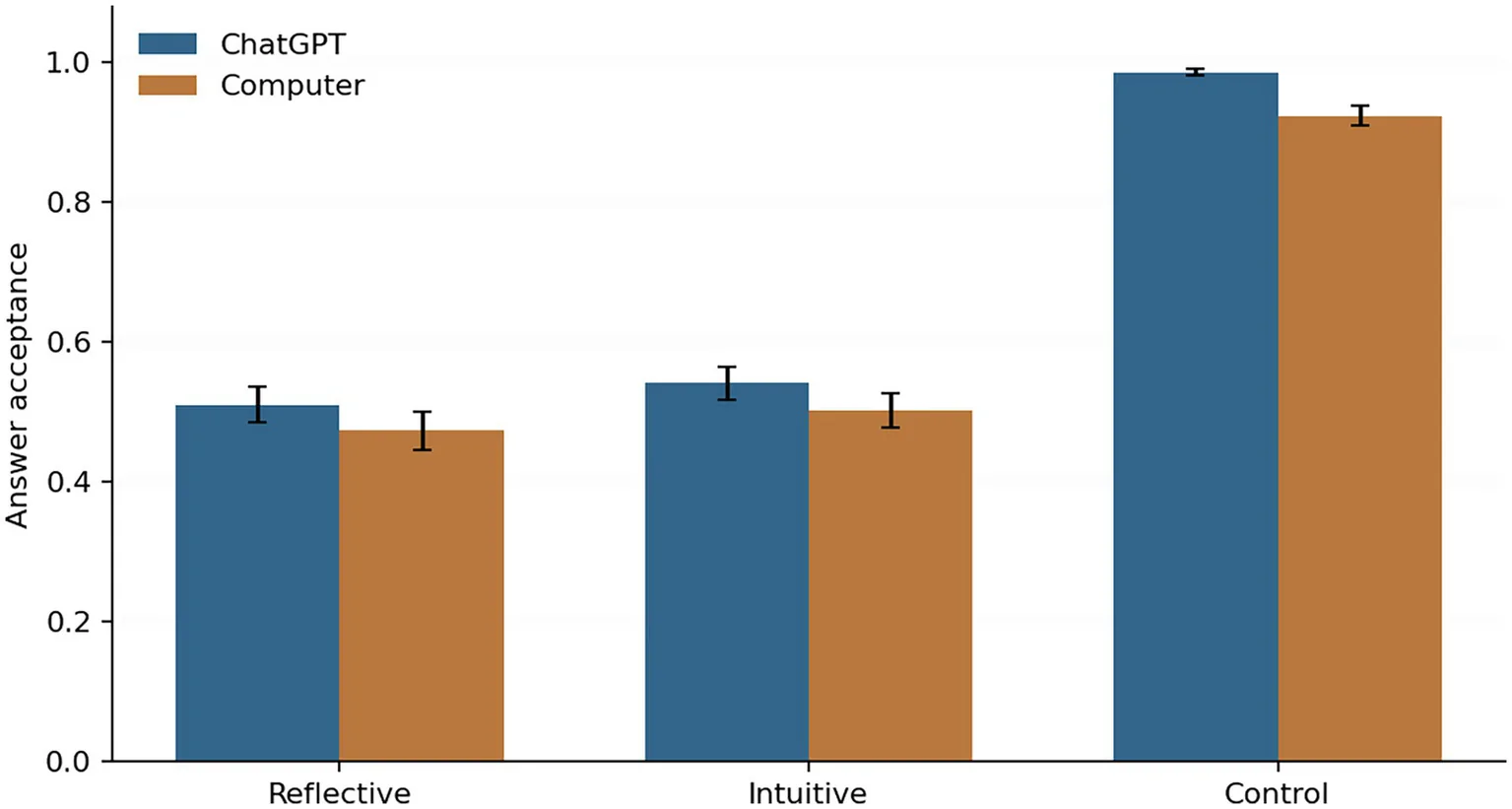
Answer acceptance by source label and answer category. Bars represent participant-level mean proportions of responses judged as correct. Error bars represent standard errors of the mean.

**Figure 3:**
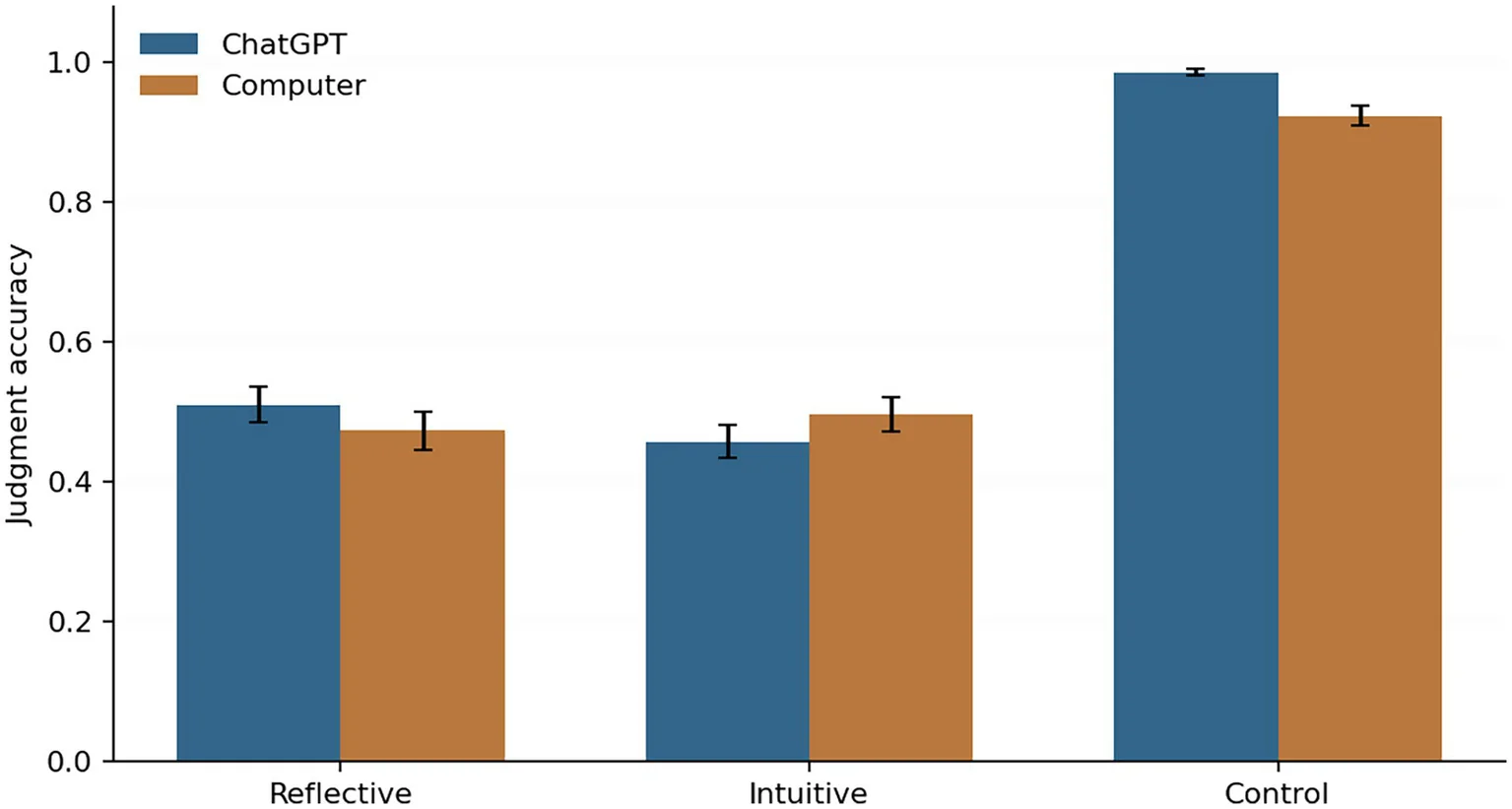
Judgment accuracy by source label and answer category. Bars represent participant-level mean proportions of accurate judgments; error bars are standard errors. Accepting a correct answer and rejecting an incorrect answer are both accurate. These values are descriptive transformations of the acceptance judgments.

**Figure 4:**
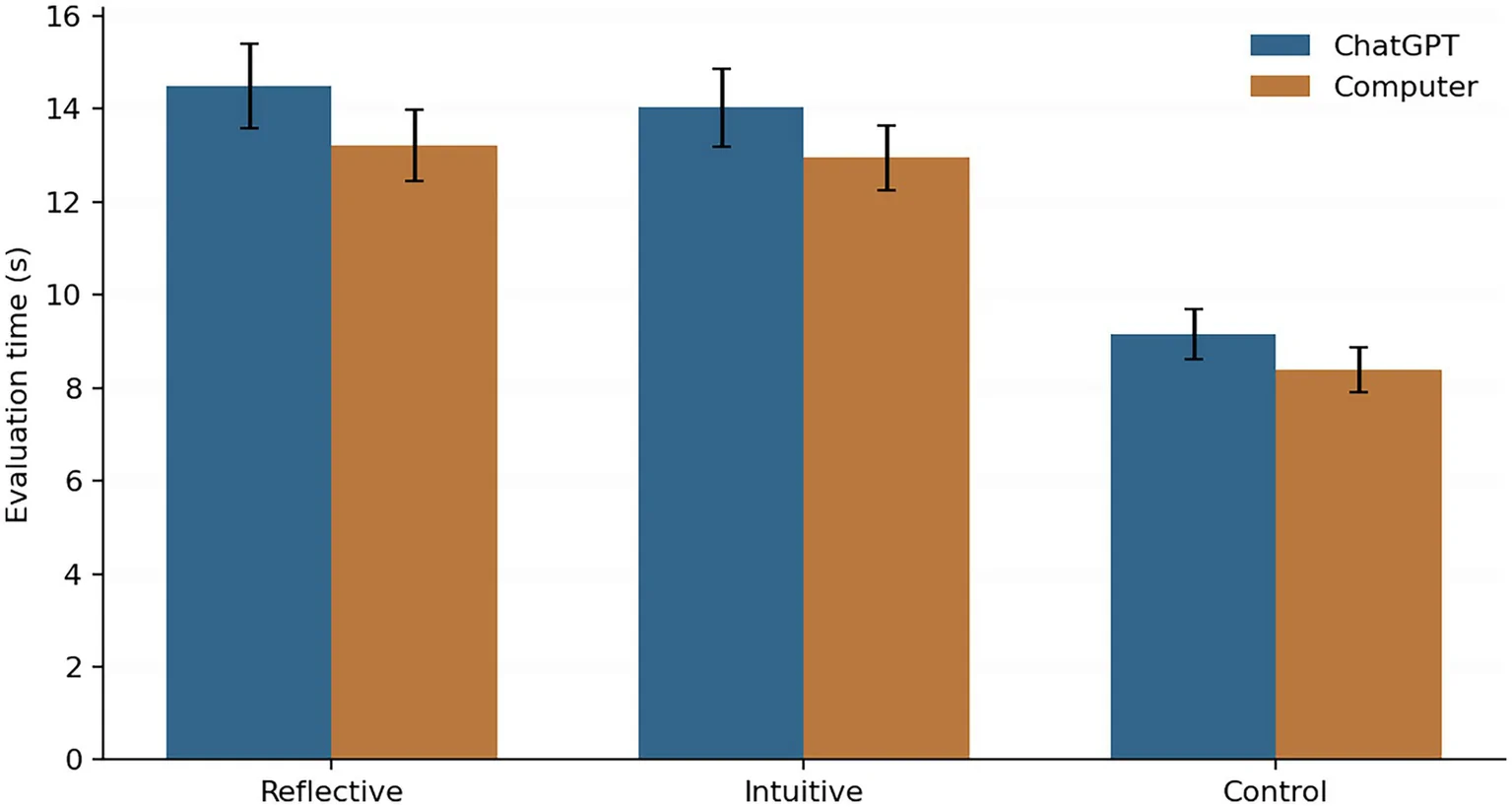
Raw mean evaluation time by source label and answer category. Bars represent participant-level mean response times in seconds. Error bars represent standard errors of the mean. Response times were screened for extremely fast responses (<300 ms) and excessively slow responses (>60,000 ms).

### Effects of source attribution on answer acceptance

The trial-level logistic mixed model showed a source-by-category interaction, likelihood-ratio *χ*^2^ (2) = 19.68, *p* < 0.001. ChatGPT attribution was associated with greater acceptance of control-correct answers, OR = 6.08, 95% CI [2.70, 13.69], *p* < 0.001. The source contrasts were not significant for intuitive-incorrect answers, OR = 1.17, 95% CI [0.92, 1.50], *p* = 0.207, or reflective-correct answers, OR = 1.17, 95% CI [0.91, 1.50], *p* = 0.227. The source association was therefore particularly pronounced for arithmetic controls.

The model converged with optimizer code 0. The participant random-intercept variance was estimated at zero, giving a singular fit; the parallel-problem-pair variance was 0.177 (SD = 0.421). The second optimizer reproduced the log likelihood and fixed effects, with a maximum coefficient difference below 0.001. Removing the participant intercept estimated at zero changed the coefficients by less than 0.000003. In the sensitivity model with an additional participant-specific source slope, its variance was also estimated at zero, and the category-specific significance conclusions were unchanged.

The participant-level acceptance ANOVA showed a main effect of Source label, *F* (1, 102) = 10.40, *p* = 0.002, partial *η*^2^ = 0.093, with more answers judged correct overall under ChatGPT (Figure 2). The category effect was significant after Greenhouse–Geisser correction, *F* (1.35, 137.96) = 159.21, *p* < 0.001, partial *η*^2^ = 0.610; Mauchly’s *W* = 0.521, *p* < 0.001, *ε* = 0.676. The source-by-category interaction was not significant in this proportion-scale analysis, *F* (2, 204) = 0.39, *p* = 0.677, partial *η*^2^ = 0.004; Mauchly’s *W* = 0.956, *p* = 0.106. The ANOVA evaluates differences in participant proportions, whereas the logistic model evaluates effects on the log-odds scale while accounting for parallel-problem pairs. Category-specific inferences are based on the trial-level model reported above. Overall acceptance was 0.680 under ChatGPT and 0.634 under Computer. The paired difference was 0.046, 95% CI [0.018, 0.074], *t* (102) = 3.225, *p* = 0.002, corresponding to approximately 4.60 percentage points.

Judgment accuracy is reported descriptively in Figure 3 and Table 2. It equals acceptance for reflective-correct and control-correct answers, and one minus acceptance for intuitive-incorrect answers. The category-specific accuracy pattern therefore follows directly from the acceptance judgments and the answer key.

### Effects of attribution on response times

The raw-time ANOVA showed a Source label effect, *F* (1, 102) = 4.61, *p* = 0.034, partial *η*^2^ = 0.043. Mean latency was 12,557.81 ms (SD = 6,557.41) under ChatGPT and 11,514.69 ms (SD = 5,567.37) under Computer (Figure 4). Greenhouse–Geisser-corrected results were *F* (1.84, 187.58) = 47.64, *p* < 0.001, partial *η*^2^ = 0.318 for category, and *F* (1.82, 185.24) = 0.22, *p* = 0.781, partial *η*^2^ = 0.002 for the interaction. Corresponding Mauchly results were *W* = 0.912, *p* = 0.010, *ε* = 0.920, and *W* = 0.899, *p* = 0.005, *ε* = 0.908. Screened trial RTs were positively skewed (skewness = 2.00); natural-log RTs had skewness = −0.03. In the log-RT analysis, the source effect was not significant, *F* (1, 102) = 1.82, *p* = 0.180, partial *η*^2^ = 0.018. The category effect remained significant, *F* (1.59, 162.35) = 56.67, *p* < 0.001, partial *η*^2^ = 0.357 (*W* = 0.743, *p* < 0.001, *ε* = 0.796). The interaction was not significant, *F* (2, 204) = 0.06, *p* = 0.939, partial *η*^2^ = 0.001 (*W* = 0.963, *p* = 0.150).

### Acceptance patterns and signal-detection analysis

Acceptance of reflective-correct answers was 0.511 under ChatGPT and 0.474 under Computer; acceptance of intuitive-incorrect answers was 0.542 and 0.503, respectively. The source differences were 0.037 and 0.039. Correct control answers were accepted at rates of 0.986 and 0.924, corresponding to rejection rates of 1.4% and 7.6%. These main-experiment endorsement rates are distinct from the unlabeled pretest, in which control judgment accuracy was 100% in both batteries.

Participant-level sensitivity was *d*′ = −0.079 (SD = 0.993) under ChatGPT and −0.071 (SD = 1.132) under Computer. The paired difference was −0.007, 95% CI [−0.176, 0.161], *t* (102) = −0.088, *p* = 0.930. Criterion was c = −0.050 (SD = 0.357) under ChatGPT and 0.033 (SD = 0.355) under Computer. The paired difference was −0.084, 95% CI [−0.178, 0.011], *t* (102) = −1.759, *p* = 0.082. Criterion was descriptively more liberal under ChatGPT; neither source comparison reached statistical significance.

In exploratory age-adjusted comparisons, the overall acceptance difference was 0.046, 95% CI [0.018, 0.074], *t* (101) = 3.23, *p* = 0.002. The raw-time difference was 1,043.12 ms, 95% CI [75.27, 2,010.96], *t* (101) = 2.14, *p* = 0.035. Thus, adjustment for age preserved the overall acceptance and raw-time results; it does not alter the separate log-time finding.

In the post-hoc control-performance sensitivity analysis, nine participants were excluded, leaving 94. Overall acceptance remained higher under ChatGPT (*M* = 0.679) than Computer (*M* = 0.640), *t* (93) = 2.571, *p* = 0.012, mean difference = 0.039, 95% CI [0.009, 0.069]. The corresponding full-sample comparison was *t* (102) = 3.225, *p* = 0.002. Mean CRT sensitivity remained close to zero in the restricted sample (ChatGPT: *d*′ = −0.123; Computer: *d*′ = −0.119), with no significant source difference, *t* (93) = −0.054, *p* = 0.957. Control rejection rates were 1.1% under ChatGPT and 3.8% under Computer.

### Individual differences

#### Exploratory individual-difference analyses

Exploratory Pearson correlations examined CTAS, HCTM, positive and negative GAAIS scores, and ChatGPT-minus-Computer differences in acceptance, judgment accuracy, and raw evaluation latency (Table 3). Positive differences indicate higher values under ChatGPT. All estimates use the corrected behavioral data and the questionnaire scoring described above.

**Table 3:** Pearson correlations among individual-difference measures and attribution-related difference scores (*N* = 103).

Variable	1	2	3	4	5	6	7
1. Critical-thinking attitude (CTAS)	–						
2. Trust in AI (HCTM)	−0.071	–					
3. Positive AI attitudes (GAAIS-P)	0.086	0.635^**^	–				
4. Negative AI attitudes (GAAIS-N)	0.032	−0.361^**^	−0.071	–			
5. Delta answer acceptance	−0.125	0.010	−0.006	0.019	–		
6. Delta judgment accuracy	0.041	0.087	0.016	−0.123	0.135	–	
7. Delta raw evaluation time	0.076	−0.008	−0.037	−0.061	−0.125	0.182	–

Delta denotes ChatGPT minus Computer. Questionnaire items were scored as described in the Methods. Judgment accuracy is derived from acceptance and the answer key. Correlations are exploratory; the acceptance and judgment-accuracy difference scores showed low internal consistency (*α* = 0.106 and −0.150, respectively). ^**^p < 0.01.

#### Trust in AI and attitudes toward AI

Trust was positively correlated with positive AI attitudes (*r* = 0.635, *p* < 0.001) and negatively correlated with negative AI attitudes (*r* = −0.361, *p* < 0.001). No significant correlations of these measures with source-related acceptance, accuracy, or latency differences were detected (Table 3).

These exploratory results describe the associations detected in the present sample; they do not establish that broad trust and AI attitudes have no role in source-related evaluation.

#### Critical thinking attitude

CTAS was not significantly correlated with the overall source difference in acceptance (*r* = −0.125, *p* = 0.207), judgment accuracy (*r* = 0.041, *p* = 0.681), or raw evaluation latency (*r* = 0.076, *p* = 0.447).

The present analyses therefore detected no significant associations between critical-thinking disposition and source-related differences. These correlations concern an evaluative disposition and should be distinguished from tests of reasoning ability or trial-level moderation.

#### Summary of individual-difference findings

The exploratory analyses detected no significant associations of the measured dispositions with source differences. Internal consistency of the overall acceptance difference score was *α* = 0.106; category-specific estimates were 0.430 for controls, −0.054 for intuitive answers, and −0.138 for reflective answers. The overall judgment-accuracy difference score had *α* = −0.150. Negative estimates reflect a lack of positive covariance among the component differences and do not support treating these scores as internally consistent individual-difference measures. The nonsignificant correlations are therefore descriptive exploratory findings and do not establish that participant characteristics are unrelated to source effects. Questionnaire internal consistency and behavioral difference-score reliability address distinct measurement properties.

## Discussion

The present study examined source-labeled answer evaluation in a modified CRT. The participant-level analysis showed greater overall acceptance under ChatGPT, while the trial-level logistic model identified a source-by-category interaction: the source association was particularly pronounced for arithmetic controls, and separate source contrasts within the two CRT categories were not significant. The independent unlabeled pretest supported battery comparability, and battery assignment and trial order were randomized. Judgment accuracy distinguishes successful evaluation from endorsement: greater acceptance can improve accuracy for correct answers and reduce it for incorrect answers. The central finding concerns source-related endorsement, with category-specific evidence supplied by the logistic model rather than an independently measured accuracy interaction.

The acceptance finding is consistent with the possibility that source identity contributes to reliance. Lee and See (2004) emphasize calibration of trust to system capability and task demands, and Dzindolet et al. (2003) demonstrate the relevance of trust to automation reliance. Logg et al. (2019) found algorithm appreciation in comparisons with human judgment. Our comparison instead involved two technological sources, a named conversational AI and a generic Computer label. Greater endorsement under ChatGPT may reflect different source expectations, but the present design does not identify which expectation explains the effect. Automation-bias research offers a related framework for reliance on system outputs (Parasuraman and Manzey, 2010).

The control-answer contrast is informative because both conditions presented objectively correct arithmetic answers in matched displays. The source cue was associated with how readily these answers were endorsed, even though evaluating them did not require resolving a CRT-style intuitive alternative. This pattern supports a contribution of attributed source identity to answer evaluation within this task. It does not identify whether participants differed in their expectations about the sources or in their verification of particular answers. The nonsignificant contrasts for CRT answers likewise do not establish that source effects are absent in those categories; their confidence intervals describe the remaining uncertainty.

The finding should also be considered alongside evidence of algorithm aversion and skepticism toward AI-labeled information. Dietvorst et al. (2015) examined reluctance to use algorithms after observing errors, whereas the present task provided fixed answers without iterative performance feedback. Longoni et al. (2022) and Altay and Gilardi (2024) found lower perceived accuracy for AI-attributed news; the latter effect also extended to true and human-created headlines. Numerical verification, a ChatGPT-specific label, and a technological comparison source distinguish the present task from those settings. Content domain, comparison baseline, and feedback are therefore plausible boundary conditions for source-label effects. These differences provide a basis for comparing source-label effects across settings.

Raw mean evaluation times were approximately one second longer under ChatGPT, but the source difference was not significant after log transformation. The latency result therefore depends on the RT summary and distributional treatment (Lo and Andrews, 2015). Scrutiny, uncertainty, attention to the label or icon, and hesitation remain possible interpretations of longer evaluation times. Processing fluency concerns ease of processing as a cue to judgment (Reber and Schwarz, 1999; Alter and Oppenheimer, 2009); because display format was matched here, it should not be treated as a manipulated explanation of the source difference. Longer elapsed time can also end in endorsement of an incorrect answer, so latency alone does not demonstrate successful verification.

The signal-detection analysis complements these findings by separating sensitivity from response criterion within CRT trials. Mean sensitivity was close to zero under both labels, and neither the sensitivity comparison nor the criterion comparison reached statistical significance (*p* = 0.930 and *p* = 0.082, respectively). Criterion was descriptively more liberal under ChatGPT. This is consistent with the absence of significant category-specific source contrasts for CRT answers in the logistic model. The overall acceptance difference, the stronger control-answer association, and CRT discrimination address distinct questions; greater overall endorsement does not establish improved discrimination or a significant criterion shift within CRT trials.

The post-hoc sensitivity analysis showed that the overall acceptance difference persisted after excluding participants with multiple arithmetic control errors. Nevertheless, CRT discrimination remained low, so this restriction did not resolve the concern about near-zero sensitivity. Acceptance of plausible supplied answers without successful verification is one possible interpretation; the present measures cannot distinguish it from misunderstanding, inattention, or other task-related factors. The persistence of the acceptance difference therefore does not establish the mechanism underlying it.

Dual-process and contextual accounts remain useful for framing why source information may contribute to answer evaluation. The contextual account of Fabio et al. (2019) emphasizes flexibility in the relation between automatic and controlled processing and environmental cues. In this task, however, answer categories describe supplied solutions, and participants’ processing modes were not measured independently. Near-zero CRT sensitivity shows that acceptance and rejection did not reliably distinguish the correct and incorrect answer sets at the group level. The pretest supports the comparability of the batteries, with high cross-battery correlations and identical perfect control performance, while its reflective accuracy was also near 0.50. The main-experiment control rejection rates and CRT sensitivity characterize different aspects of performance, distinguishing straightforward arithmetic verification from evaluation of CRT-derived answers.

The second aim concerned individual differences. Trust and general AI attitudes were interrelated, while their correlations with source differences were not significant; the same was true of critical-thinking disposition. Internal consistency of the acceptance and accuracy difference scores was low, constraining interpretation of these associations. The absence of significant correlations cannot therefore be taken as evidence that source-related evaluation operates independently of trust, AI attitudes, or critical-thinking disposition. These findings describe the exploratory associations observed in this sample.

Taken together, the findings show greater overall answer acceptance under ChatGPT and a particularly pronounced association for arithmetic controls in the trial-level model. The pretest and randomized assignment support the comparison of the two source conditions. Distinguishing overall endorsement, category-specific source contrasts, judgment accuracy, SDT sensitivity and criterion, and evaluation latency provides a coherent account of the behavioral findings.

## Limitations and future directions

Convenience recruitment and the sample’s educational and occupational composition limit generalization to populations with different numerical expertise or AI experience. The broad age range also permits heterogeneity, although exploratory age adjustment preserved the overall acceptance result. Five trials per source-by-category cell provide a brief assessment; more trials would improve the precision of participant proportions and individual-difference estimates. The observed low internal consistency of behavioral difference scores further constrains interpretation of the exploratory correlations. The correlation sensitivity benchmark indicates that modest associations may remain undetected.

Answer category and objective truth were not fully crossed: reflective and control answers were correct, whereas intuitive answers were incorrect. Category differences therefore combine answer properties and validity. Evaluating supplied answers also differs from generating solutions in the classic CRT. The source cue included both a label and a small icon, and attribution was symbolic rather than part of live chatbot interaction. The Computer comparison may evoke expectations of arithmetic reliability, so the result does not establish how either source would compare with a human or unlabeled baseline.

A source manipulation check, ChatGPT familiarity, and prior CRT exposure were not assessed. Salient source cues may also contribute to demand characteristics in a within-subjects task. Future studies could assess these expectations, increase item counts, and cross answer validity with content category. Confidence ratings and more direct process measures could help distinguish verification, uncertainty, and attention to source information.

The arithmetic controls were not dedicated attention checks. Moreover, the post-hoc exclusion criterion used an outcome that itself varied with source attribution. The restricted-sample results should therefore be treated as a sensitivity analysis rather than evidence that the source effect is independent of inattentive responding or misunderstanding.

## Conclusion

The present study shows greater overall acceptance of answers attributed to ChatGPT than to Computer in an evaluative reasoning task. The trial-level logistic model indicates that the source association was particularly pronounced for arithmetic controls; source contrasts within the reflective-correct and intuitive-incorrect CRT categories were not significant. Pretested parallel batteries were randomly assigned to source labels, and trial order was randomized.

Judgment accuracy clarifies whether participants appropriately accept a correct answer or reject an incorrect one. SDT detected no significant source difference in sensitivity or criterion, although criterion was descriptively more liberal under ChatGPT. The source difference in raw mean evaluation times was not significant after log transformation. No significant exploratory associations with the measured dispositions were detected. Together, these results distinguish source-related endorsement from discrimination and from assumptions about underlying processing modes.

Understanding how AI labels influence evaluative judgment is essential as generative AI systems become increasingly embedded in contexts where accuracy, verification, and critical evaluation carry significant personal and societal consequences.

## Data Availability

The raw data supporting the conclusions of this article will be made available by the authors, without undue reservation.
