# Supplementary material for: The ChatGPT source cue shapes answer acceptance in a reasoning task
Source: Front Psychol. 2026 Oct 23;17:1923236. doi: 10.3389/fpsyg.2026.1923236 (PMC13739812; doi:10.3389/fpsyg.2026.1923236)
Supplement: Supplementary file 1 [file Supplementary_file_1.docx]

The ChatGPT Source Cue Shapes Answer Acceptance in a Reasoning Task

**Supplementary Material**

**List of CRT and Control Items Used in the Experimental Task**

All items consisted of a problem paired with a pre-generated response, which participants evaluated as correct or incorrect. All responses were prepared in advance by the researchers and displayed with either the ChatGPT or Computer source label. Computer denotes the generic technological comparison label rather than an identified live software system. Each participant evaluated 30 answers, with 15 per source and five per source within each category. Assignment of the two batteries to source labels was randomized between participants, and trial presentation order was randomized. Each problem was presented once.

Items below are grouped by answer category for presentation. The numbers 1–30 are catalog numbers in this supplement; they do not identify the source shown to an individual participant, the randomized trial sequence, or the source-specific dataset columns. The analytical pairing follows corresponding dataset positions across the two source conditions, as described in the manuscript. No source assignment or presentation order should be inferred from this list.

**A. CRT Items - Reflective Correct Response**

Intuitive and reflective responses do not coincide; the provided response is reflective and correct.

| **Item** | **Problem** | **Pre-generated response** | **Objective status of response** |
| --- | --- | --- | --- |
| 1 | An eraser and a pen cost €2.10 in total. The pen costs €2.00 more than the eraser. How much does the eraser cost? | €0.05 | Correct |
| 2 | Ten students write ten introductions in ten minutes. How long would 100 students take to write 100 introductions? | 10 minutes | Correct |
| 3 | Conference attendees double every day. If the conference hall is full on day 98, on which day is it half full? | Day 97 | Correct |
| 4 | Five bakers knead five loaves of bread in five minutes. How many loaves do ten bakers knead in ten minutes? | 20 | Correct |
| 5 | On a farm there are 40 heads and 94 legs. Some animals have two legs and some have four. How many rabbits are there? | 7 | Correct |
| 6 | If five teachers give five lessons in five hours, how long does it take one teacher to give one lesson? | 5 hours | Correct |
| 7 | A car and a trailer cost €31,000 in total. The car costs €30,000 more than the trailer. How much does the trailer cost? | €500 | Correct |
| 8 | In a shoe rack there are 10 tennis shoes and 10 formal shoes, all unpaired. In the dark, how many shoes must you take to be sure of having at least one pair of the same type? | 3 | Correct |
| 9 | A bracelet costs €100. The jeweler offers a 50% discount and then increases the price by 50%. What is the final price? | €75 | Correct |
| 10 | Twenty factories produce twenty machines in twenty hours. How long would 100 factories take to produce 100 machines? | 20 hours | Correct |

**B. CRT Items - Intuitive Incorrect Response**

Intuitive and reflective responses do not coincide; the provided response is intuitive and incorrect.

| **Item** | **Problem** | **Pre-generated response** | **Objective status of response** |
| --- | --- | --- | --- |
| 11 | A bat and a ball cost €1.10 in total. The bat costs €1.00 more than the ball. How much does the ball cost? | €0.10 | Incorrect |
| 12 | Five machines produce five parts in five minutes. How long would it take 100 machines to produce 100 parts? | 100 minutes | Incorrect |
| 13 | Water lilies double in size every day. If the lake is completely covered in 48 days, on which day is it half covered? | 24 days | Incorrect |
| 14 | Four machines produce four T-shirts in four minutes. How many T-shirts do eight machines produce in eight minutes? | 8 | Incorrect |
| 15 | On a farm there are 35 heads and 94 legs. How many rabbits are there? | 23 | Incorrect |
| 16 | If 10 workers complete 10 puzzles in 10 hours, how long does it take one worker to complete one puzzle? | 1 hour | Incorrect |
| 17 | A dress and a hat cost €250 in total. The dress costs €200 more than the hat. How much does the hat cost? | €50 | Incorrect |
| 18 | In a drawer there are 10 black socks and 10 white socks, all unpaired. In the dark, how many socks must you take to be sure of having at least one pair of the same color? | 2 | Incorrect |
| 19 | An item costs €100. The store offers a 50% discount and then increases the price by 50%. What is the final price? | €100 | Incorrect |
| 20 | Three ovens bake three loaves of bread in three minutes. How long would six ovens take to bake six loaves? | 6 minutes | Incorrect |

**C. Control Items - Non-CRT**

Simple arithmetic or transparent reasoning items without intuitive-reflective conflict; the provided response is correct.

| **Item** | **Problem** | **Pre-generated response** | **Objective status of response** |
| --- | --- | --- | --- |
| 21 | You have 3 apples and buy 4 more. How many apples do you have in total? | 7 | Correct |
| 22 | How many fingers do two hands have in total? | 10 | Correct |
| 23 | If 6 cookies are divided equally among 3 children, how many cookies does each child receive? | 2 | Correct |
| 24 | In a picture there are 2 cats and 3 dogs. How many animals are there in total? | 5 | Correct |
| 25 | What number comes after 12? | 13 | Correct |
| 26 | If you are on the third floor and go down two floors, which floor are you on? | First floor | Correct |
| 27 | If a class starts at 3:00 p.m. and lasts 1 hour, what time does it end? | 4:00 p.m. | Correct |
| 28 | If you have a €1 coin and a €2 coin, how much money do you have in total? | €3 | Correct |
| 29 | A child has 5 balloons and loses 2. How many balloons remain? | 3 | Correct |
| 30 | There are 10 pens in a box. If you remove 4, how many pens are left? | 6 | Correct |

# D. Trial-level source comparisons

The binomial logistic mixed model included source, answer category, and their interaction, with crossed random intercepts for participants and the 15 parallel-problem pairs. All 3,090 judgments from 103 participants were included. Maximum-likelihood estimation used a Laplace approximation in R 4.3.3 with lme4 1.1-35.1. The source-by-category interaction was significant, likelihood-ratio χ²(2) = 19.68, p < .001.

Table S4. Source contrasts for answer acceptance

| Answer category | ChatGPT acceptance | Computer acceptance | OR [95% CI] | p |
| --- | --- | --- | --- | --- |
| Control-correct | 98.64% | 92.43% | 6.08 [2.70, 13.69] | < .001 |
| Intuitive-incorrect | 54.17% | 50.29% | 1.17 [0.92, 1.50] | .207 |
| Reflective-correct | 51.07% | 47.38% | 1.17 [0.91, 1.50] | .227 |

Note. Acceptance percentages are observed rates. Odds ratios compare ChatGPT with Computer within each category and are estimated from the logistic mixed model. Confidence intervals are Wald intervals; p values are unadjusted and two-sided. Holm adjustment across the three contrasts did not change the significance conclusions. The participant-intercept variance was estimated at zero; pair variance was .177. Both optimizers converged, and sensitivity checks for the random-effect structure preserved the conclusions.
